# A Comprehensive Review of Indel Detection Methods for Identification of Zebrafish Knockout Mutants Generated by Genome-Editing Nucleases

**DOI:** 10.3390/genes13050857

**Published:** 2022-05-11

**Authors:** Blake Carrington, Kevin Bishop, Raman Sood

**Affiliations:** Zebrafish Core, National Human Genome Research Institute, National Institutes of Health, Bethesda, MD 20892, USA; carringb@mail.nih.gov (B.C.); bishopk@mail.nih.gov (K.B.)

**Keywords:** zebrafish, CRISPR/Cas9, indels, HMA, fluorescent PCR, qPCR, HRMA, AS-PCR, RFLP, LDR

## Abstract

The use of zebrafish in functional genomics and disease modeling has become popular due to the ease of targeted mutagenesis with genome editing nucleases, i.e., zinc finger nucleases (ZFNs), transcription activator-like effector nucleases (TALENs), and clustered regularly interspaced short palindromic repeats/Cas9 (CRISPR/Cas9). These nucleases, specifically CRISPR/Cas9, are routinely used to generate gene knockout mutants by causing a double stranded break at the desired site in the target gene and selecting for frameshift insertions or deletions (indels) caused by the errors during the repair process. Thus, a variety of methods have been developed to identify fish with indels during the process of mutant generation and phenotypic analysis. These methods range from PCR and gel-based low-throughput methods to high-throughput methods requiring specific reagents and/or equipment. Here, we provide a comprehensive review of currently used indel detection methods in zebrafish. By discussing the molecular basis for each method as well as their pros and cons, we hope that this review will serve as a comprehensive resource for zebrafish researchers, allowing them to choose the most appropriate method depending upon their budget, access to required equipment and the throughput needs of the projects.

## 1. Introduction

Zebrafish (*Danio rerio*) are a powerful model organism for functional genomics and human disease modeling as ~71% of human genes have at least one conserved zebrafish ortholog and most perform similar functions [1]. Furthermore, ~82% of human disease associated genes have a zebrafish ortholog [1]. Prior to the discovery of genome editing nucleases, functional genomics and disease modeling in zebrafish was performed by random mutagenesis followed by forward and reverse genetic approaches [2,3,4,5,6,7,8,9,10]. Advances in genome editing tools, i.e., ZFNs, TALENs, and CRISPR/Cas9, have allowed zebrafish researchers to selectively target genes of interest for functional studies. These genome editing nucleases function by creating a double stranded break (DSB) at the desired site in the genome, leading to activation of the cell’s endogenous repair pathways. Repair of DSBs by error-prone non-homologous end joining (NHEJ) pathway results in indels at the target site [11]. On average, two out of three indels lead to loss-of-function frameshift mutations [12] that can be used to establish gene knockout zebrafish models. In addition to ZFNs, TALENs and CRISPR/Cas9, CRISPR/Cas12a (also known as Cpf1) has recently been shown to work in zebrafish. It allows users to target regions not targetable by Cas9 [13,14,15,16,17].

In recent years, CRISPR/Cas9 has become the genome editing nuclease of choice due to its ease of design and synthesis of single guide RNAs (sgRNAs) for a target site [12,18,19,20]. Thus, high throughput protocols have been developed to generate single or multiple gene knockout zebrafish models using CRISPR/Cas9 [21,22,23,24,25]. A typical workflow to generate and characterize gene knockout mutants using CRISPR/Cas9 involves the following steps: (1) The design and synthesis of target-specific sgRNAs; (2) Microinjections into 1-cell embryos; (3) Somatic analysis in injected embryos (optional); (4) Founder screening to identify germline transmitting founders with desired indels; (5) Fin biopsies of progeny from selected founders to establish stable lines; and (6) Phenotype evaluation and genotype-phenotype correlations [12,26,27,28,29]. In cases where an alternate genome-editing nuclease (ZFN, TALEN or CRISPR/Cpf1) is chosen for a gene knockout project, the workflow is the same, except for the first step. The availability of efficient methods for identifying fish with indels induced by these genome-editing nucleases is critical to the success of mutant generation. Thus, a variety of methods have been developed or adopted from previously developed methods for the identification of single nucleotide polymorphisms (SNPs) for indel identification. While some methods work for both somatic and germline analysis, others are specifically designed for either somatic or germline analysis. Therefore, an appropriate method needs to be selected based on the project requirements. Somatic analysis is performed in injected fish, which are mosaic for a variety of indels, to determine the target-specific activity of the nuclease before proceeding to generate stable lines, or to evaluate phenotypes in the founder generation (termed as “crispant”) [30,31,32,33,34,35,36]. Germline analysis is performed during founder screening and genotyping for line maintenance and phenotype analysis.

The goal of this review is to provide an overview of the indel detection methods currently used in the zebrafish. Specifically, we have provided details about their molecular basis, equipment requirements, sensitivity, and limitations. In general, these methods involve DNA extraction from embryos (individual or pooled depending upon the application) or fin biopsies followed by amplification of the indel-containing region with standard, fluorescently labeled or allele-specific primers. Genomic DNA can be extracted using commercially available reagents/kits or solutions prepared in the laboratory. We routinely use a Extract-N-Amp Tissue PCR kit (Sigma) or HotSHOT method using NaOH and Tris-HCl as described [24,37,38]. A 96-well plate containing zebrafish embryos or fin biopsies can be processed in half an hour with either of these methods and DNA is used directly or after 1:10 dilution as a template for PCR [24,37,38]. It is best to optimize PCR primers and conditions using DNA from WT samples for robust amplification of the target region. Indels are detected by either the direct analysis of PCR products or the post processing of PCR products by sequencing or enzymatic digestion. Thus, we have grouped various methods that fit each of these categories to make it easier for the reader to select an appropriate method for their needs.

## 2. Methods Based on Direct Analysis of PCR Products

### 2.1. Indel Detection by Polyacrylamide Gel Electrophoresis (PAGE) of PCR Products

**Figure 1:**
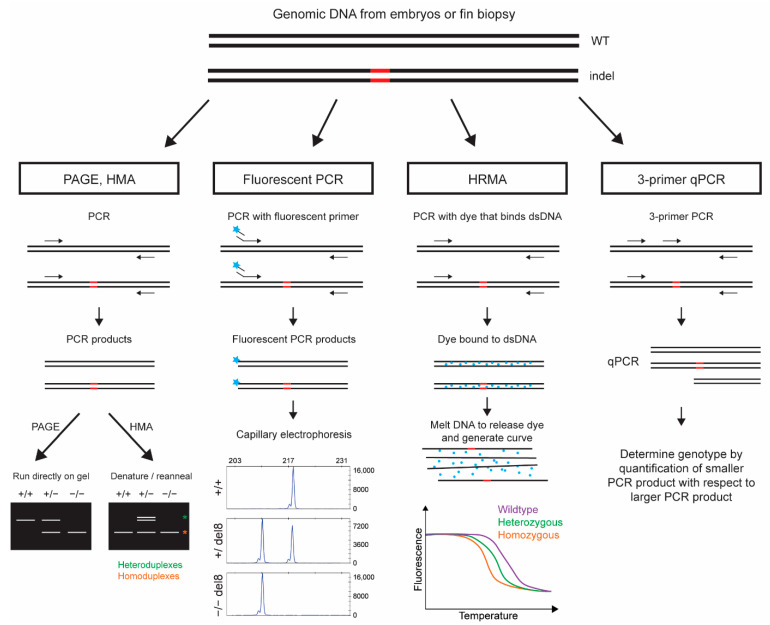
A schematic depicting main steps and output for methods that identify indels by direct analysis of PCR products. Genomic DNA containing an indel (marked in red) is PCR amplified and directly analyzed using either of the methods listed here. PAGE is straightforward with size separation of PCR products by running on polyacrylamide gels. HMA is performed by denaturation and reannealing of PCR products to form homo- and/or heteroduplexes which are separated by PAGE gels. Fluorescent PCR involves use of a fluorescently labeled primer and PCR products are run on a capillary electrophoresis instrument. In HRMA, PCR is performed in the presence of a fluorescent dye that binds dsDNA. As the PCR products melt the dye is released and fluorescence is measured, resulting in a melt curve which corresponds to the genotype. A 3-primer qPCR is performed with primers that flank the indel site with a third primer that overlaps the indel site. PCR is performed on a qPCR instrument and the relative quantity of the smaller PCR product is compared to the larger PCR product to determine the genotypes in the sample.

Direct analysis of PCR products by size separation on polyacrylamide gels is the most straightforward method to screen for indels as demonstrated by its successful use in zebrafish [39]. However, PAGE has a limiting resolution of accurately detecting smaller indels. Detection of 1 bp insertions or deletions is only possible if the amplicon size is smaller than 126 bp [39], in which case larger indels can be missed. Technically, PAGE can be used for somatic analysis and founder screening, as well as genotyping by looking for a smear for mosaic embryos and discrete bands for founder screening and genotyping. However, this method is best suited for the genotyping of known indel alleles by the optimization of amplicon size and the percent of polyacrylamide for gels (Figure 1). Overall, this method is a low throughput and labor-intensive method as it involves the loading and imaging of multiple gels, especially if used during the phenotype analysis of established mutants.

### 2.2. Indel Detection by Heteroduplex Mobility Assay (HMA)

Indel detection by HMA is based on the fact that the heteroduplexes migrate slower than the homoduplexes in a gel due to the presence of a bulge in the mismatch region. In this assay, PCR products are denatured by heating and allowed to reanneal by cooling slowly. If indels exist in a sample, both homo- and heteroduplexes are formed during the reannealing process. Reannealed PCR products are analyzed by polyacrylamide or agarose gels (Figure 1). Its successful use in zebrafish has been demonstrated for the detection of ZFN, TALEN and CRISPR- induced indels in injected fish and F1 progeny during founder screening [40,41,42]. However, since both wild-type (WT) and homozygous mutant samples run as homoduplexes, a modified method called mixing HMA (mHMA) was developed for genotyping [43]. mHMA requires that homoduplex samples be run on a gel a second time after mixing them 1:1 with a known WT sample, followed by heating and reannealing. This mixing leads to heteroduplex formation in the homozygous mutant sample but not in the WT samples, enabling the user to differentiate WT samples from homozygous mutant samples. Another limitation of the HMA method is the resolution of indel size, especially if agarose gels are used for analysis. Indels smaller than 3 bp in size can be missed as the bulge is not large enough to cause a detectable change in the migration of heteroduplex DNA compared to homoduplex DNA [44]. Therefore, a modified version of HMA, termed PRIMA (Probe-Induced HMA) was recently developed to overcome the limited resolution of mutation size that can be detected by HMA [44]. Specifically, the focus of PRIMA is the accurate genotyping of samples with 1 bp insertions or deletions. They also replaced gels with a microchip electrophoresis system (MultiNA) to make it an easier and high-throughput assay. To our knowledge, HMA and its modified versions are not commonly used by zebrafish investigators, likely due to the labor involved in running gels or the ability to gain access to the MultiNA equipment.

### 2.3. Indel Detection by Fluorescent PCR and Capillary Electrophoresis

In this method, the target region is amplified in the presence of a fluorescently labeled primer, followed by fragment separation and sizing on a capillary electrophoresis instrument (i.e., sequencing machines). Our laboratory was the first to develop this method for the genotyping of zebrafish with indels using a 3-primer mixture for fluorescent labeling of the amplicons [38]. The primer mixture consists of a fluorescently labeled (6-FAM or HEX) M13F primer, an amplicon specific forward primer with M13F tail and an amplicon specific reverse primer with a PIG tail (5′-GTGTCTT) to ensure that PCR products are adenylated and of uniform size [45]. Subsequently, modified versions of our method have been developed. Yang and colleagues [46] substituted a fluorescently labeled M13F primer with a universal primer and termed their method IDAA (Indel Detection by Amplicon Analysis). Ramlee and colleagues [47] used a traditional 2-primer PCR with a fluorescently labeled forward primer. The main advantage of using a 3-primer mixture is to cut the costs associated with the fluorescent labeling of forward primers for all amplicons and is best suited for investigators involved in the generation and analysis of several gene knockouts. Irrespective of the method used to generate fluorescent PCR products, further analysis is performed by adding a size standard to the PCR product and fragment separation on a capillary electrophoresis instrument. Fragment size plots are analyzed with software programs such as Genemapper (ThermoFisher) or Peak Studio [48] to accurately size all detected fragments in a sample with 1 bp resolution (Figure 1).

Despite the high costs associated with the setup of a capillary electrophoresis instrument, fluorescent PCR is a popular genotyping method in zebrafish as it is fast, accurate and allows to scale up for analysis of large numbers of samples during genotype-phenotype correlation experiments [12,21,23,27,32,49,50,51,52,53,54,55,56,57,58,59,60,61]. Fluorescent PCR is also a versatile method as it allows the analysis of single gene knockouts [21,23,38,50,51,52,53,54,56,57,60,61,62], multiple gene knockouts using the MultiFRAGing approach [63], or injected embryos using CRISPR-STAT (Somatic Tissue Activity Test) for evaluation of CRISPR cutting efficiency [30,32,34,64,65,66,67,68]. A few commercial vendors (Eurofins Genomics, GENEWIZ) offer fragment analysis services as an option for laboratories that do not have access to a sequencing machine. However, it might be cost-prohibitive to outsource the genotyping of a large number of samples as is often required for most zebrafish projects.

### 2.4. Indel Detection by High Resolution Melting Analysis (HRMA)

HRMA is another highly sensitive and high throughput method frequently used in zebrafish to assess indels in somatic tissues, as well as for the genotyping of established mutants [69,70,71,72,73]. This method is based on the differences in melting temperature of double stranded DNA for WT, vs. mutant alleles that can be measured by quantification of fluorescent dye bound to double-stranded DNA using specific equipment (i.e., Lightscanner or Lightcycler 480). The assay begins with PCR amplification of the target region in the presence of a double stranded DNA binding dye (i.e., EvaGreen, LC Green or Chromofy) that does not inhibit PCR and emits a fluorescent signal upon binding to double stranded DNA. After PCR, samples are heated and as specific duplexes denature dye is released, leading to a drop in the fluorescent signal, thus generating a melt curve which is plotted as the level of fluorescence (*y*-axis) vs. the temperature (*x*-axis). Each allele within a sample generates its own melt curve and these melt curves can be compared to assign genotypes (Figure 1).

An important consideration for HRMA experimental design is to optimize primers and PCR conditions such that each allele within the sample is amplified without bias. Therefore, it is recommended to use online tools [74,75] for the design of primers and prediction of melt curves for accurate genotyping. In general, smaller amplicons yield a larger difference in the melting temperatures. It has been demonstrated that the ideal size of amplicon for HRMA in zebrafish is 100 bp, thus limiting the use of HRMA for detecting larger indels [73]. Another limitation of the HRMA method is that single nucleotide polymorphisms (SNPs) can also alter melting curves [71,73]. Thus, care must be taken when designing amplicons to exclude endogenous polymorphisms which frequently occur in the zebrafish genome [76] as they can complicate data analysis by altering melt curves.

HRMA can be performed in 96 or 384 well plates, making it a high throughput genotyping assay. It is also highly sensitive, as demonstrated by an evaluation of CRISPR/Cas9 activity in injected embryos as early as 2-cells [72] and detection of chimerism as low as 5% in founder zebrafish [73]. The main drawback of using HRMA by most laboratories is the initial cost for equipment setup. D’Agostin [69] and colleagues addressed this issue with modifications to run HRMA analysis on a Real-Time PCR system used for qPCR that is readily available in most laboratories and cheaper to setup. However, its sensitivity and specificity decreases as they found that indels under 15 bp were not always distinguishable from the WT allele.

### 2.5. Indel Detection by 3-Primer Quantitative (q)PCR

A 3-primer quantitative PCR based system was developed for germline indel screening in an F1 zebrafish progeny [77]. In this assay, an additional internal forward primer is designed to overlap the putative indel site based on the site of CRISPR-induced DSB. qPCR is performed as a single reaction with two forward primers and a common reverse primer. Thus, two PCR products of different sizes are generated from WT samples, whereas the indel disrupts the binding site of the internal forward primer, resulting in the loss of the smaller PCR product in mutant samples. Genotypes are determined by quantification of the smaller and larger size PCR products relative to each other (Figure 1). Although there are no reports of its use for somatic analysis, it is possible to evaluate injected embryos for the presence of indels by a change in the ratio of the PCR products, compared to the WT embryos.

## 3. Methods Based on Analysis of Post Processing PCR Products

### 3.1. Indel Detection by Sanger Sequencing

Sanger sequencing is one of the high throughput methods for indel detection and is straightforward to perform with access to a capillary electrophoresis instrument and commercially available sequencing kits. PCR products are sequenced using the same primers as used for PCR set-up and resulting chromatograms are analyzed to identify indels using a WT sequence as a reference. Although the initial cost of equipment set-up is expensive, many institutes have set-up sequencing core facilities and samples can also be sent out to commercial sources that perform Sanger sequencing. This method can be used for indel detection during somatic or germline analysis as described below.

#### 3.1.1. Somatic Analysis by Deconvolution of Sanger Sequence Reads

**Figure 2:**
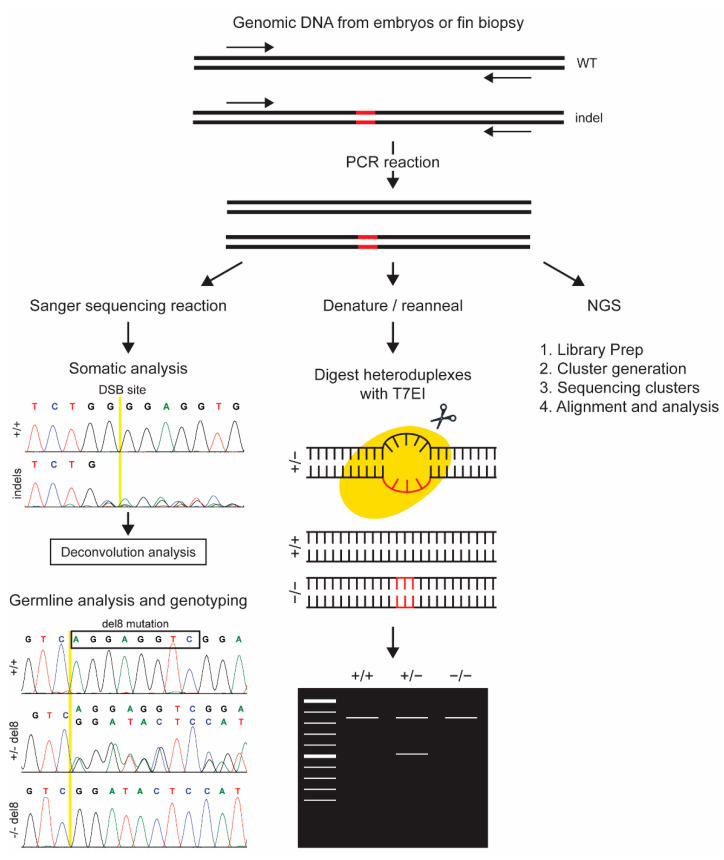
A schematic depicting main steps and output for indel detection methods that require further processing of PCR products before analysis. Sanger sequencing can be used for analysis of both somatic and germline samples followed by either manual or computational analysis of chromatograms. T7EI assay is performed by denaturation and reannealing of PCR products followed by T7EI digestion. Heteroduplexes are digested with T7EI while homoduplexes stay intact. Digested products are run on a gel and heterozygous samples can be differentiated while WT and homozygous sample run at the same size and cannot be distinguished from each other. Next generation sequencing (NGS) is carried out with a kit for library preparation, cluster generation, and sequencing of clusters. Sequence reads are then aligned and analyzed with bioinformatics tools.

The analysis of somatic tissues such as injected embryos is a common practice for the assessment of the target specific activity of the designed nuclease. Traditionally, this is achieved by the cloning of PCR products from injected embryos followed by the sequencing of multiple clones to accurately assess mutation profiles [22,36,38,62]. Alternatively, to avoid cloning and save time, PCR products from the injected embryos are directly sequenced. However, the resulting chromatograms are difficult to interpret visually as multiple bases are called at the same position due to a wide variety of indels (Figure 2). Thus, computational tools such as Tracking of Insertions and Deletions (TIDE) [78], CRISP-ID [79], Deconvolution of Complex DNA Repair (DECODR) [80] and Inference of CRISPR Edits (ICE) [81] have been developed to deconvolute these types of data. Each tool runs a unique algorithm on chromatograms from a WT and a genome edited sample and provides an output file with the predicted indels. Further validation by cloning may be required depending on the intended application. CRISP-ID has limitations for somatic analysis as only a single chromatogram can be analyzed at a time and the output is limited to three indels per sample. Although the other methods can analyze multiple chromatograms simultaneously and output is not limited, the output data still need manual curation, making somatic analysis by Sanger sequencing a low throughput method.

#### 3.1.2. Sanger Sequencing for Germline Indel Detection and Genotyping

The use of Sanger sequencing for the interpretation of germline indels is straightforward compared to the somatic analysis as there are only two possible alleles in a germline sample. Although sequence of the mutant alleles can be deduced by the manual analysis of chromatograms from a heterozygous sample as demonstrated by Varshney and colleagues [24], it becomes challenging to infer long or complex indels. Therefore, online tools have been developed to make this process easier. Poly Peak Parser [82] and CRISP-ID [79] are online tools that compare Sanger sequence data from a sample with germline indels to a WT reference sequence and provide the sequence of the mutant allele as an output. A similar function can be performed using MS Word using the SWS strategy (Sanger sequencing and the Wildcard Search function) [83]. Poly Peak Parser seems to be the preferred method among zebrafish users [84,85,86,87,88]. Once zebrafish lines with desired mutant alleles are established, samples can be easily genotyped by observing chromatograms for clean WT or homozygous mutant reads, or double peaks indicating heterozygous samples (Figure 2).

### 3.2. Indel Detection by T7 Endonuclease I (T7EI) Digestion Assay

In this assay, T7 endonuclease I is used to specifically cleave regions of mismatches in DNA. Like HMA, PCR products are denatured and slowly reannealed to generate homo- and/or heteroduplexes. These duplexes are then digested with T7EI followed by gel electrophoresis to separate the digested products from the undigested full-length products (Figure 2). T7EI assay has been shown to work well for indel detection in zebrafish both in somatic tissues and in F1 samples to determine if indel mutations are transmitted to the progeny [20,69,89,90,91]. However, T7E1 assay cannot be used for the genotyping of established mutant lines since it will not distinguish WT from homozygous mutant samples, as both genotypes only generate homoduplexes which do not undergo digestion with T7EI. An important consideration in designing T7EI assay is to make sure that the amplicon does not contain any endogenous SNPs which could lead to a false positive result. Although Surveyor assay (using CelI enzyme) functions in a similar way [92], it has not been popular among zebrafish investigators due to high costs associated with its procurement from the proprietary source (Transgenomic).

### 3.3. Next Generation Sequencing (NGS)

Illumina or Ion Torrent based NGS is used for the deep sequencing of PCR amplicons through a four-step process: (1) library preparation, (2) cluster generation, (3) sequencing, and (4) alignment and data analysis (Figure 2). During the library preparation step, PCR products are labeled with an adapter (i.e., barcodes). The library prep is then attached to a slide or flow cell and amplified to generate clusters. These clusters are then sequenced by synthesis using fluorescently labeled terminated nucleotides (Illumina) or by measuring the release of hydrogen ions via semiconductors when nucleotides are washed over the slide (Ion Torrent). Following sequencing, reads can be separated and clustered based on barcodes that were added during the sample preparation for further analysis. Since the NGS method provides high depth of sequencing coverage, it is often used to screen somatic activity in injected embryos [22,34,36,66,93,94]. However, the analysis of NGS data is challenging and requires familiarity with computational and bioinformatic tools. In addition, many small labs do not have easy access to NGS equipment and using core facilities or commercial vendors may be expensive.

## 4. Indel Detection Methods That Require the Mutant Allele to Be Known

### 4.1. Indel Detection by Allele-Specific PCR (AS-PCR) Based Assays

**Figure 3:**
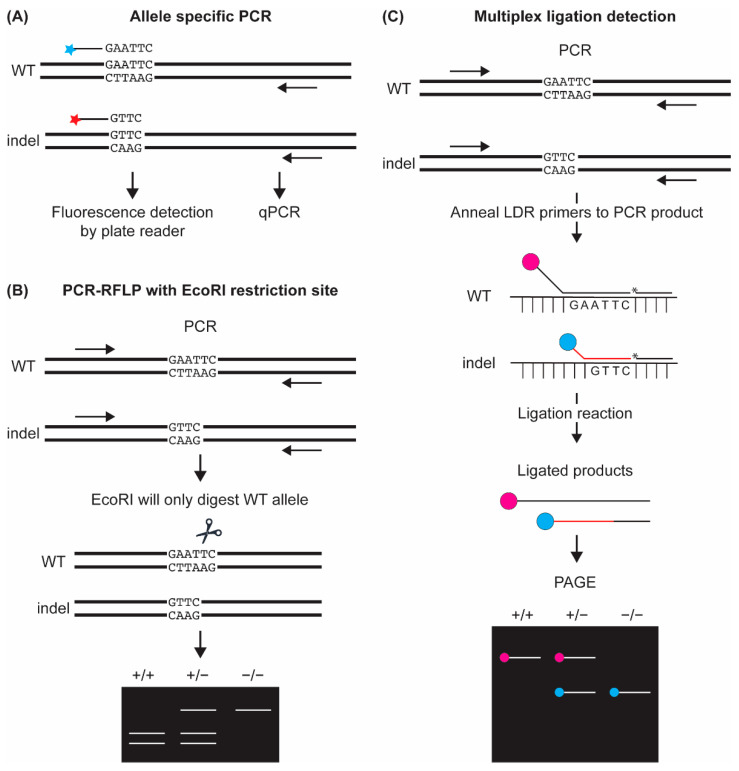
Indel detection methods that require the mutant allele to be known. We used a 2 bp deletion (delAA) as an example to illustrate these methods in this figure. (**A**) Allele-specific PCR (AS-PCR) is performed using forward primers that are allele specific and each has a unique fluorophore (red and blue stars). Fluorescence can be detected following the PCR with a plate reader or in real time with a qPCR instrument. (**B**) RFLP assay is conducted by digestion of PCR products with a restriction enzyme that will only recognize the WT or mutant allele. After digestion samples are run on a gel and genotypes can be determined based on bands which correlate to if digestion occurred or not. (**C**) Multiplex ligation detection is carried out post-PCR amplification. LDR primers consisting of two allele-specific primers and a common primer are annealed to the PCR products. Primers are then ligated together if the common primer and allele specific primer are perfectly adjected to each other. The ligated products are run on a PAGE gel to determine genotypes. Red and blue circles denote different fluorophores used for WT and mutant alleles.

Several variations of AS-PCR assays have been developed to efficiently genotype zebrafish lines with known indel mutations, i.e., after lines with the desired indel mutations are selected for further investigation. Typically, an AS-PCR assay involves amplification of the target region using two forward primers and one common reverse primer. The forward primers are designed such that their 3′ ends are unique and match either the WT or the indel sequence (Figure 3). To incorporate a fluorescent signal, either the forward primers are labeled at their 5′ ends with different fluorophores or universal fluorescent probes are used to avoid the costs associated with the labeling of forward primers for each locus (Figure 3). Fluorescence quenchers are added to eliminate noise due to nonspecific fluorescent signals. For detection of the fluorescent signal, either a qPCR instrument is used, or PCR products are analyzed using a plate reader. The commonly used AS-PCR assays in zebrafish are: allele-specific qPCR (ASQ) [95] and the KBioscience Competitive Allele-Specific PCR (KASP) genotyping assay [93]. While ASQ can be performed by anyone with access to a qPCR instrument, KASP technology is proprietary (LGC Genomics), requiring the manufacturer to design primers for the target region and provide all reagents for running the assay. The main advantage of AS-PCR based assays is their high throughput as they do not require the post-PCR processing of samples or running of gels.

### 4.2. Indel Detection by PCR-Restriction Fragment Length Polymorphism (PCR-RFLP) Assay

A PCR-RFLP assay is based on the distinction between WT and indel sequences by the presence or absence of a specific restriction site. Thus, if the mutant sequence leads to the loss or creation of a specific restriction site, fragments of different lengths can be generated by the digestion of PCR products with the chosen restriction enzyme [95,96,97,98,99,100]. Digested products are run on agarose gels to determine the genotype of the samples based on the size of DNA fragments (Figure 3). Although PCR-RFLP assay is easier to design after an indel allele that disrupts or creates a restriction site is chosen for further analysis, it has been also used in somatic analysis and founder screening by designing the nuclease such that the predicted DSB site overlaps with a restriction enzyme recognition site [97,98,99,100,101,102,103,104]. Despite being a straightforward method, the PCR-RFLP method is limited by the fact that the indel may not always lead to a loss or gain of a restriction site and its low throughput due to the labor involved in the loading and imaging of gels.

### 4.3. Indel Detection by Multiplex Ligation Detection Assay

Another version of an allele specific assay demonstrated to work in zebrafish is the adapter PCR ligation detection reaction (PCR-LDR) [105]. In this protocol, PCR is followed by an LDR reaction using two allele-specific primers and a common primer, termed as LDR primers. The allele-specific primers are designed with different length tails and/or fluorescent tag modifications, one for the WT sequence and one for the mutant allele. A common primer is designed from the region adjacent to the allele-specific primers and is phosphorylated at the 5′ end. Following a PCR of the target region, a protease digestion reaction is performed to remove any polymerase from the reaction and the LDR primers are annealed to PCR products. DNA ligase is then used to ligate the primers together if they are perfectly adjacent to each other. These ligated primer products are run on a PAGE gel to determine genotypes (Figure 3). While PCR-LDR can be multiplexed using tails of different sizes or different fluorescent tags, analysis is slow and labor intensive as all samples need to be run on PAGE gels.

## 5. Conclusions

**Figure 4:**
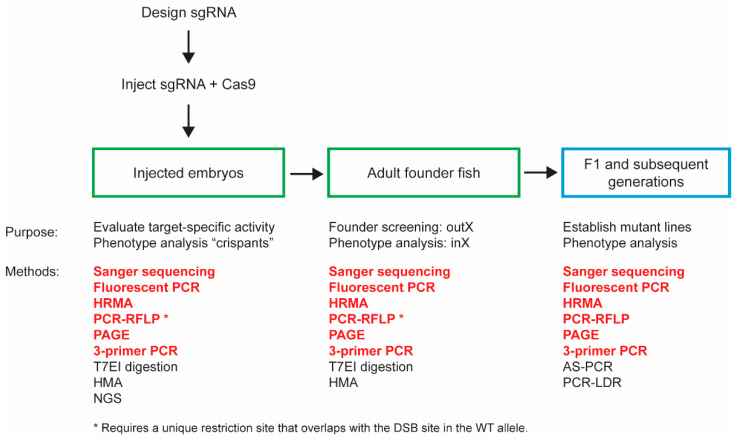
A schematic of the workflow to establish and characterize indel mutants with the applicable indel detection methods for somatic analysis, founder screening or genotyping listed underneath. Green boxes represent when indels are not known while the blue box is when an indel mutation has been established. Methods that can be used at all steps of mutant generation and characterization are highlighted in red.

In summary, a variety of methods have been developed to identify fish with indels during the process of mutant generation and phenotype analysis (Figure 4). In this review, we have provided brief descriptions of all currently used assays with the hope of providing a resource to the zebrafish community. Overall, indel detection methods applicable to zebrafish range from labor-intensive low throughput methods (such as running PCR products directly, after heteroduplex formation or enzymatic digestion on polyacrylamide gels), to high throughput methods that are amenable to automation (such as fluorescent PCR, HRMA, sequencing and qPCR). In addition, allele-specific PCR based assays can be designed once a desired indel mutation is chosen for further analysis. In Figure 4, we have summarized the steps involved in mutant generation and characterization with a list of all the applicable methods for each step. A final word of caution in choosing and designing the assay is to avoid SNPs in the PCR amplicons, specifically in the region used for primer design and in the entire amplicon for methods that use heteroduplex formation, i.e., HMA, HRMA, T7EI assay and PCR-RFLP assay. In Table 1, we have provided a quick overview of specific requirements and limitations for each method, listing high throughput methods first. A quick glance at this table should help readers in choosing the best method for their projects. It is worth mentioning that these indel detection methods should work irrespective of the genome-editing nuclease used. Thus, zebrafish lines generated by ZFNs and TALENs can be genotyped using any of these methods. Furthermore, the indel detection methods discussed in this review can be used for similar applications in other model organisms, e.g., in mice or cultured cells. The only requirement is that genomic DNA can be extracted to amplify the target region. We believe that this review will help zebrafish investigators choose the most suitable method for indel detection based on their needs and accessibility to equipment.

## Figures and Tables

**Table 1:** List of throughput, specific requirements, and limitations for each indel detection method.

Method	Throughput	Specific Requirements		Limitations
		Equipment	Reagents *	
Fluorescent PCR	High	• Capillary electrophoresis instrument	• Fluorescently labeled primers • Size standard (e.g., ROX400)	• Access to equipment
HRMA	High	• Lightscanner or qPCR instrument	• dsDNA binding dye	• Amplicon size • Access to equipment • Sensitive to SNPs in amplicon
3-primer qPCR	High	• qPCR instrument	• qPCR reagents	• Access to equipment
NGS	High	• Illumina or Ion Torrent instrument	• Appropriate NGS kit	• Data analysis • Expensive • Access to equipment • Cannot be used for founder screening or genotyping
AS-PCR	High	• Plate reader or qPCR instrument	• Depends on chosen platform	• Cannot be used for somatic analysis or founder screening • Access to equipment
Sanger sequencing	Low/High **	• Capillary electrophoresis instrument	• Post-PCR clean up kit • Dye terminator kit • Sequence reaction cleanup kit	• Data analysis • Expensive • Access to equipment
PAGE	Low	• None	• Polyacrylamide gels	• Laborious • Resolution of smaller indels
HMA	Low	• None	• Polyacrylamide gels	• Laborious • Resolution of smaller indels • Sensitive to SNPs in amplicon
T7EI digestion	Low	• None	• Post-PCR clean up kit • T7 endonuclease I • Gels	• Laborious • Sensitive to SNPs in amplicon • Cannot be used for genotyping
PCR-RFLP	Low	• None	• Post-PCR clean up kit • Restriction enzyme • Gels	• Requirement for indel to cause loss or creation of a unique restriction site
PCR-LDR	Low	• Gel imager capable of detecting fluorescence	• LDR primers • Protease • DNA ligase • Polyacrylamide gels	• Laborious • Access to equipment

* Reagents for DNA extraction and PCR not included as they are required for all methods except NGS. ** Low throughput for somatic analysis; high throughput for founder screening and genotyping.

## Data Availability

Not applicable.
